# Brompton and Harefield Infection Score (BHIS): A novel scoring system for detection and treatment of sternal wound infection.

**DOI:** 10.1186/1749-8090-10-S1-A12

**Published:** 2015-12-16

**Authors:** Maria Cannoletta, Alan Soo, Melissa Rochon, Feriel Mahiout, Anthony de Souza, Rashmi Yadav

**Affiliations:** 1 Department of Cardiac Surgery, Royal Brompton Hospital, London, UK

## Background/Introduction

Sternal wound infection (SWI) is a significant complication that increases risk of mortality, prolongs hospital stay, and increases healthcare cost. In our institution, we developed a novel scoring system (Brompton & Harefield Infection Score - BHIS) to identify patients at high risk of developing SWI following cardiac surgery with sternotomy. Patients identified to have high BHIS score was then treated with prophylactic intervention package.

## Aims/Objectives

We report our results of this novel intervention.

## Method

The BHIS score is scored based on variables including female gender (2), diabetes (1) or HbA1c =>7.5 (3), BMI >35 (2) and LVEF <45% (1). Patients were stratified into low risk (BHIS score 0-1), medium risk (BHIS score 2-3) and high risk (BHIS score 4 and above).

An intervention package was then developed for patients identified to have high BHIS score risk. The intervention package includes extended antimicrobial prophylaxis, standardized sternal closure, support wear (females), negative pressure dressing and patient education.

## Results

Data was collected between November 2013 and May 2015. During this period, 811 patients underwent cardiac surgery requiring sternotomy. In the low risk group, 9 patients out of 542 patients (SWI rate 1.7 per 100 operations) developed SWI. In the medium risk group, 7 patients out of 210 patients (SWI rate 3.3 per 100 operations) developed SWI. In the high risk group (59 patients), 20 patients received prophylactic intervention. In this group, there was no SWI, whereas in the 39 patients who did not receive prophylactic intervention, there was 5 SWI (SWI rate 12.8 per 100 operations).

## Discussion/Conclusion

The BHIS score is a novel scoring system for identifying patients at high risk of SWI. Patients identified to have high BHIS score and subsequently treated with prophylactic intervention are found to have reduced SWI rate.

